# Assessment of a consensus definition of obesity and metabolic health phenotypes in children at different pubertal stages

**DOI:** 10.1038/s41598-022-25771-5

**Published:** 2022-12-07

**Authors:** Ana Pereira, Marcela Reyes, Camila Corvalán, Juan Pablo Espejo, Verónica Mericq, Mariana Cifuentes

**Affiliations:** 1 Institute of Nutrition and Food Technology (INTA), Universidad de Chile, El Libano Macul, 5524 Santiago, Chile; 2 Institute of Maternal and Child Research, Faculty of Medicine, University of Chile, Santiago, Chile; 3 Advanced Center for Chronic Diseases (ACCDiS), Santiago, Chile

**Keywords:** Biomarkers, Risk factors, Health care

## Abstract

Not all individuals with obesity develop metabolic complications, which has brought about the concepts of metabolically healthy and unhealthy obesity (MHO/MUO). However, inconsistent definitions of these conditions have limited their understanding. We assessed whether a recently-proposed consensus definition for MHO/MUO correlates with adiposity and reflects metabolic risk parameters during puberty. Low-middle income children from the Growth and Obesity Cohort Study (Santiago, Chile) were included (n = 949; 1692 visits at Tanner (T)2, T4 and/or one-year post menarche (1YPM)). Anthropometry, body composition and metabolic parameters were compared between MUO and MHO, and also in children without obesity. The risk for presenting MUO phenotype was significantly elevated with higher waist-height ratio (T2), zBMI (T2, T4), trunk fat, and C-reactive protein (T4). Elevated cardiometabolic indices were important predictors of the “unhealthy” phenotype allocation in children with or without obesity. Our observations suggest that the consensus definition in children at T2, T4 and 1YPM reflects metabolic risk and central obesity. Metabolic health phenotype allocation by this equation enables easy detection of risk factors that call for action to prevent long-term metabolic derangements in children with obesity and, importantly, also those without obesity.

## Introduction

Obesity research in adults and in children has shown that its definition based on body mass index (BMI) includes a remarkably wide inter-individual variability in cardiovascular and metabolic health. Although a single definition does not yet exist, the concept of “metabolically healthy obesity” (MHO) describes the condition of BMI-defined obesity with proper metabolic health as opposed to the classic “metabolically unhealthy obesity” (MUO). A prevalence up to 70% of MHO has been reported, depending on the definition used and population studied^1^. Even though the concept is controversial^2–4^, several studies have shown the persistence of a proportion of subjects with obesity in the healthy track^5,6^. Furthermore, physiological differences, genetic and metabolomic determinants in MHO versus MUO strongly support its validity^7–9^. As mentioned, there is currently no single definition for MHO, which has impeded the unified interpretation of different studies, led to conflicting results and hindered the advancement of this field.

Even though MHO and MUO have received less attention in children than in adults, studies in young populations have also highlighted the difficulties derived from heterogeneous MHO definitions^10,11^. In 2018, Damanhoury et al.^12^ proposed a consensus-based definition of MHO with potential universal value for comparisons between studies and clinical decision-making for children with obesity^12^.

On the other hand, there is one other less-regarded but high-risk phenotype: the non-obese but metabolically unhealthy (MUNO). These subjects have been also defined as “normal weight obese”^13^, as they do not meet the obesity criterion according to BMI; however, they show elevated total fat and cardiovascular risk. Due to the absence of evident obesity, MUNO children may go unnoticed and untreated for years, increasing the risk for cardiometabolic disease and death in adulthood^14^. Thus, valid and accepted MHO and MUNO definitions in children are needed to identify candidates for age-appropriate interventions according to their cardiometabolic health risk, which is of great relevance for decisions in public health system resource allocation and for clinical obesity management^15^. In this context, our aim was to apply the consensus equation proposed by Damanhoury et al.^12^ at different pubertal time points (Tanner 2 [T2], Tanner 4 [T4] and girls 1-year post-menarche [1YPM]) in the Growth and Obesity Cohort Study, and assess its association with the total amount and distribution of adiposity, metabolic parameters and indices. We further explored these associations in children without obesity.

## Subjects and methods

### Participants

The study is set up in the Growth and Obesity Cohort Study (GOCS) in Santiago, Chile, an ongoing cohort of 1190 children recruited in 2006 at age 4, from different day care centers from the South East Area of Santiago (low-middle socioeconomic level). The cohort was originally established to assess the association of early postnatal growth and timing of adiposity rebound in preschool-aged children. The inclusion criteria comprised full-term birth, birth weight > 2500 g and < 4500 g, and no diagnosed genetic or medical conditions that could affect growth^16^. We have routinely collected anthropometric, clinical and body composition information as well as blood samples at T2 and/or T4 and/or 1YPM in a subsample of girls (further details of data collection has been reported previously)^17^. Children with or without obesity and complete information available to calculate their metabolic and nutritional status based on Damanhoury’s^12^ consensus formula (described in “Computed indices” section) were included (n = 949 children, with 1692 visits with complete information).

The study was approved by the Ethics Committee of the Institute of Nutrition and Food Technology at the University of Chile (Resolution N°15/2021). Informed consent was obtained from all parents or guardians and the children provided their assent before starting data collection. All methods were performed in accordance with the relevant guidelines and regulations. The current study follows the guidelines of the STROBE checklist to ensure its quality (https://www.strobe-statement.org/checklists).

### Anthropometry, body composition and clinical assessments

Weight, height, hip circumference (HC) and waist circumference (WC) were measured by a trained nutritionist with standardized procedures. Weight was registered to the nearest 10 g with minimal clothing (underwear) on a portable electronic scale with capacity to 200 kg and accuracy to 10 g (Seca 770, SECA, Hamburg, Germany). Height was measured to the nearest 0.1 cm using a portable Holtain stadiometer (Harpenden 603; Holtain LTD, Crosswell, United Kingdom). Participants were positioned with the heels touching the stadiometer wall plate, head erect, and arms to their sides. With participants standing, WC (cm) was measured at the minimum circumference between the iliac crest and the rib cage, and HC (cm) was measured at the level of the greater trochanter using a metal, self-locking tape measure with accuracy to 0.1 cm (Lufkin W606 PM; Cooper Tools, Raleigh, NC). The measurements were taken twice, with a third measurement if the difference between the first two exceeded 0.3 kg for weight or 0.5 cm for height, WC or HC. The intra-observer technical error of measurement and mean observer bias were within the limits suggested by the World Health Organization in the Growth Reference Study^18^. Body mass index (BMI) was calculated as weight (kg) divided by the height (m) squared. Standard z-scores of weight for age, height for age, and BMI for age were estimated based on the World Health Organization^19,20^. WC divided by height or hip circumference were used to calculate the waist/height ratio (WHtR) and waist/hip ratio (WHipR), respectively. Central obesity was defined as WC ≥ 90th centile of hispanics, according to a study in Mexican–American children and adolescents^21^. We also report subjects presenting percentile ≥ 75 for central adiposity based on the same study.

Body fat and fat-free mass (FFM) percentages were assessed at each visit using Tanita-BC-418 MA bioelectrical impedance (BIA) measurements (Tanita-Corporation, Tokyo, Japan), at a measurement frequency of 50 kHz (accuracy 0.1 kg). Fat mass index (FMI) was calculated as total fat mass (FM, kg) divided by the height (m) squared, whereas fat-free mass index (FFMI) was calculated by dividing fat-free mass (kg) by height (m) squared (all based on data from BIA). Additionally, the “fat to lean ratio” or “load capacity model”^22^ was calculated based on BIA as FM (kg) divided by FFM (kg) and by FM (kg) divided by FFM squared (kg^2^). The percentage of total and truncal body fat as well as lean soft tissue were also evaluated at T4 by dual-energy X-ray absorptiometry (DXA), using Ghc Lunar Prodigy DPX-NT (Lunar Radiology, Madison, WI). This scanner estimates total body lean soft tissue and fat mass during a 5-min sweep^23^. Subjects were measured in a supine position on the evaluation bed, wearing undergarments and covered with a robe.

Blood pressure measurement was performed using a digital automatic monitor (Omron 705 IT) on the right arm, with the lower edge of the sleeve 1 inch above the elbow, after the participants had been resting for at least 10 min. Four measurements for every participant with a 1 min minimum time interval were taken. If the difference between the assessments exceeded 10 mmHg, the procedure was repeated, and the mean value of the second, third and fourth measurements was used for analysis. Age- and sex-specific 90th percentiles for systolic and diastolic blood pressure were determined according to Flynn et al.^24^.

### Puberty

Since 2010, visits at INTA were performed every 6 months to assess pubertal maturation using Tanner stages based on breast in girls (by visual inspection and palpation)^25^ and on genitalia in boys (by inspection and palpation using the Prader orchidometer)^26^. Assessments were performed by a trained dietitian and supervised by a pediatric endocrinologist. Breast development was assessed with inter and intra-observer concordance > 0.9^27^. After girls reached Tanner stage 3, the date of the first bleeding (age at menarche) was asked at every visit. Boys’ genitalia were evaluated with an inter-observer concordance of Cohen’s kappa = 0.8 (testes volume of 3 or 4 ml)^28^.

### Metabolic and hormone assessments and calculations

Fasting blood samples were obtained (10 ml), centrifuged, and serum samples were stored at − 80 °C until further processing for measuring circulating metabolites and hormones. Samples were analyzed at the Nutrition Laboratory of the Catholic University Medical Center (Santiago, Chile). This laboratory conducts daily assessments of the accuracy of the measurements using quality control softwares (Bio-Rad Laboratories Inc., Hercules, CA), and has a Certificate of Traceability periodically updated by the Centers for Disease Control and Prevention (CDC). Serum glucose was measured using enzymatic colorimetric techniques (HUMAN; Gesellschaft für Diagnose und Biochemical, Wiesbaden, Germany) and serum insulin was measured with a radioimmunoassay kit (Linco Research Inc., St. Charles, MO). Total cholesterol (T-Chol) and triglycerides (TG) were measured using enzymatic colorimetric techniques (HUMAN). High-density lipoprotein cholesterol (HDL) was isolated by precipitation with a solution of sodium phosphotungstate magnesium chloride^29^. LDL cholesterol was calculated using the Friedewald formula (i.e., all concentrations of triglycerides were < 400 mg/dl)^30^. Serum leptin and adiponectin were measured using commercial radioimmunoassays (Millipore) and C-reactive protein (CRP) was assessed with a high-sensitivity (hs-) enzyme immunoassay kit (BIOMERICA, Inc.).

### Computed indices

We used the Damanhoury et al.^12^ consensus formula to determine the MHO and MUO groups, and based on these criteria we also defined metabolically healthy and unhealthy groups among non-obese children (MHNO and MUNO, respectively). According to Damanhoury’s definition^12^, metabolically healthy subjects meet all of the following conditions: HDL > 40 mg/dl (> 1.03 mmol/l), TG ≤ 150 mg/dl (≤ 1.7 mmol/l), systolic (SBP) and diastolic (DBP) blood pressure ≤ 90th percentile, and a measure of “normal” glycemia. The authors’ definition lacked consensus regarding the measure of glycemia, and since most of the studies used fasting glycemia ≤ 100 mg/dl (≤ 5.6 mmol/l), we used this same parameter. Obesity was defined as BMI-for-age (zBMI) > 2 SDs above the WHO Growth Reference median (97th percentile).

The Homeostatic Model Assessment for Insulin Resistance (HOMA-IR) index was calculated as fasting glucose (mmol/l) × fasting insulin (mU/ml)/22.5.

The Visceral Adiposity Index (VAI), a sex-specific model to estimate metabolic risk based on anthropometric and lipid profiles^31^, was calculated as follows:

$${\text{Boys:}}{\text{ VAI }} = \, ({\text{WC}}\left( {{\text{cm}}} \right)/({39}.{68} + \left( {{1}.{88}*{\text{BMI}}} \right) \, *\left( {{\text{TG}}/{1}.0{3}} \right) \, *\left( {{1}.{31}/{\text{HDL}}} \right)$$

$${\text{Girls:}}{\text{ VAI }} = \, ({\text{WC}}\left( {{\text{cm}}} \right)/ \, ({36}.{58} + \left( {{\text{BMI }}*{1}.{89}} \right) \, *\left( {{\text{TG}}/0.{81}} \right) \, *\left( {{1}.{52}/{\text{ HDL}}} \right)$$

The triglyceride-glucose index (TyG), a low-cost assessment method shown to be useful for estimating the risk for insulin resistance, type 2 diabetes, metabolic syndrome and cardiovascular diseases in children, adolescents and adults^32,33^ was obtained by using the following formula^34^:

$${\text{Ln [fasting \; triglycerides \; (mg/dl)}} \times {\text{fasting}}\,{\text{glucose}}\,{\text{(mg/dl)/2]}}$$

### Statistical analysis

We used two-way ANOVA to describe and compare the parameters involved in group allocation among phenotypes (MHO-MUO-MHNO-MUNO). Bonferroni post-hoc analysis was used to assess pairwise differences when an obesity*metabolic health interaction was observed. Independent sample t-tests were used to assess differences in the clinical and biochemical characteristics between children allocated to the MHO vs. MUO and MHNO vs. MUNO groups at T2, T4, and 1YPM (subgroup of girls). To identify determinants for the unhealthy phenotypes (MUO and MUNO), logistic regression was performed, in age- and sex- adjusted models to estimate the Odds Ratio (OR) and 95% confidence intervals (95%CI). p-values < 0.05 were considered statistically significant. Statistical analysis was performed using Stata version 16.0 (Stata Corp LLC, TX, USA).

## Results

### Sample description

The original cohort consisted of 888 children in T2 (51% males), 808 in T4 (52% males) and 361 girls at 1YPM. Children without complete data to define metabolic health were excluded, namely 29%, 11% and 4% for T2, T4 and 1YPM, respectively. Supplementary Fig. S1 shows a diagram of the number of participants included and excluded for each pubertal stage. Supplementary Table S1 compares sex, age and zBMI between included and excluded children (due to incomplete data) with and without obesity. At T2 we lost more girls than boys and younger children; at T4 we observed no significant differences between included and excluded children and at 1YPM included girls were younger than those excluded. There were no differences in zBMI between included and excluded children at any of the pubertal stages.

Supplementary Fig. S2 shows a Venn diagram of the number of participants in each phenotype at each pubertal stage. Children in T2 included 141 subjects with obesity (72% male), 52% of which were allocated to the metabolically healthy groups, and 488 children without obesity (52% male), 59% of which were allocated to the healthy phenotypes. At T4, there were 118 subjects with obesity (57% male) and 599 without obesity (51% male), with 39% and 62% allocated to the healthy phenotypes, respectively. Data were available for 346 girls at 1YPM, of which 17% had obesity. Among girls with obesity, 49% were classified as healthy, while the healthy phenotype was observed in 70% of those without obesity. The mean values of the parameters used for phenotype allocation^12^ at T2, T4 and 1YPM are described in Supplementary Tables S2, S3 and S4, respectively. Age, sex and the number and percent of MUO and MUNO subjects with alterations in each of the formula’s parameters is shown in Table 1. At T2, 41% of the MUO participants were diagnosed as such with 2 or more altered factors, while this was only 17% in the MUNO (i.e., 83% of MUNO children had only one altered factor). At T4 it was 31% and 11% and at 1YPM 23% and 10% respectively. In the 3 pubertal stages, the most frequent alterations in MUO were HDL, TG and SBP, while in MUNO HDL appears as the most critical parameter, particularly at T4 and girls at 1YPM.

**Table 1:** Participants description and altered parameters from the Damanhoury formula in MUO and MUNO children at Tanner 2, Tanner 4 and 1-year post menarche.

	Tanner2				Tanner 4				1 year post menarche			
	MUO	MHO	MUNO	MHNO	MUO	MHO	MUNO	MHNO	MUO	MHO	MUNO	MHNO
N (%)	68 (10.8)	73 (11.6)	199 (31.6)	289 (46.0)	72 (10.0)	46 (6.4)	227 (31.7)	372 (51,9)	30 (8.7)	29 (8.4)	87 (25.1)	200 (57.8)
Male Sex (%)	47 (69.1)	54 (74.0)	111 (55.8)	141 (48.8)	46 (63.9)	21 (45,7)	119 (52.4)	186 (50)	–	–	–	–
Age (mean ± SD)	10.8 ± 1.1	11.1 ± 1.2	10.9 ± 1.1	10.9 ± 1.2	12.6 ± 1.6	12.0 ± 1.5	12.6 ± 1.5	12.5 ± 1.5	12.8 ± 1.0	12.8 ± 1.1	12.9 ± 1.0	13.1 ± 1.0
**Altered parameters**	n (%)		n (%)		n (%)		n (%)		n (%)		n (%)	
≥ 2 altered factors	28 (41.2)	–	34 (17.1)	–	22 (30.6)	–	24 (10.6)	–	7 (23.3)	–	9 (10.3)	
Glycemia > 100 mg/dl	14 (20.6)	–	29 (14.6)	–	7 (9.7)	–	12 (7.5)	–	3 (10)	–	5 (5.8)	
HDL ≤ 40 mg/dl	30 (44.1)	–	91 (45.7)	–	26 (36.1)	–	124 (54.6)	–	7 (23.3)	–	56 (64.4)	
TG > 150 mg/dl	26 (38.2)	–	45 (22.6)	–	32 (44.4)	–	75 (33.0)	–	16 (53.3)	–	25 (28.7)	
SBP > p90	20 (36.8)	–	60 (30.2)	–	32 (44.4)	–	37 (16.3)	–	12 (40)	–	9 (10.3)	
DBP > p90	5 (7.4)		9 (4.5)		2 (2.8)		2 (0.3)		0 (0)	–	1 (1.2)	

MUO, metabolically unhealthy obese; MHO, metabolically healthy obese; MUNO, metabolically unhealthy non-obese; MHNO, metabolically healthy non-obese; SPB, systolic blood pressure; DBP, diastolic blood pressure; TG, triglycerides; HDL-C, high-density lipoprotein cholesterol.

### Metabolically healthy and unhealthy obesity

#### Anthropometric and body composition variables

At T2 (n = 141, Table 2), age-adjusted BMI and waist/height ratio (WhtR) were 6% and 2% lower, respectively in children classified as MHO as compared with those MUO (p < 0.05). More differences in anthropometric variables were revealed upon analysis at T4 (n = 118, Table 2). Adolescents allocated to the healthy phenotype showed an 8% lower age-adjusted BMI, 4% lower hip circumference, 5% lower waist circumference, 12% lower absolute fat-free mass and 8% lower FFMI. The fat to lean ratio (“load capacity model”^22^ FM/FFM or FM/FFM2) or central obesity as defined by age- and sex- adjusted WC ≥ 90th percentile did not differ between healthy and unhealthy subjects. With DXA data available for this visit, MHO subjects also showed 10% lower total fat, 14% lower trunk fat, and 4% lower percent trunk fat with no differences in percent total fat. Available data for girls at 1YPM showed no differences in anthropometry or body composition between obesity phenotypes (Table 2).

**Table 2:** Description of adiposity, metabolic and hormonal markers in participants of the GOCS Study with obesity at Tanner 2, Tanner 4 and 1 year postmenarche.

	Tanner 2		Tanner 4		1 year post menarche	
	MUO (68)	MHO (73)	MUO (72)	MHO (46 )	MUO (30)	MHO (29)
**Anthropometry**						
WC (cm)	85.3 ± 7.6	84.4 ± 7.3	91.16 ± 8.74	86.83 ± 7.06**	90.6 ± 9.6	89.8 ± 6.8
WC ≥ 90 (n)	45 (66.2%)	45 (61.6%)	50 (69.4%)	28 (60.1%)	21 (70.0%)	20 (69.0%)
WC ≥ p75 (n)	68 (100%)	73 (100%)	71 (98.6%)	46 (100%)	30 (100%)	29 (100%)
HC (cm)	91.6 ± 6.8	91.4 ± 6.5	100.10 ± 7.29	96.39 ± 6.86**	105.7 ± 10.6	104.6 ± 7.2
zBMI	2.63 ± 0.47	2.46 ± 0.36*	2.51 ± 0.42	2.30 ± 0.22**	2.48 ± 0.68	2.44 ± 0.31
WHipR	0.93 ± 0.05	0.92 ± 0.04	0.91 ± 0.05	0.90 ± 0.05	0.86 ± 0.04	0.86 ± 0.04
WhtR	0.58 ± 0.04	0.57 ± 0.04*	0.57 ± 0.04	0.56 ± 0.03	0.57 ± 0.06	0.57 ± 0.03
**Body composition**						
FFM (%)^†^	66.2 ± 4.8	65.9 ± 4.8	68.1 ± 4.5	68.0 ± 5.6	63.1 ± 4.5	61.8 ± 4.8
FFM (Kg)^†^	36.4 ± 5.7	36.3 ± 5.8	48.86 ± 9.61	42.80 ± 8.07***	44.36 ± 4.92	43.44 ± 4.49
Total Fat (%)^†^	34.0 ± 4.4	33.8 ± 4.4	31.78 ± 4.65	31.53 ± 4.79	36.92 ± 4.47	38.25 ± 4.78
Total Fat (kg)^†^	19.1 ± 5.4	18.9 ± 5.5	22.97 ± 6.48	20.49 ± 7.43	26.45 ± 7.35	27.43 ± 6.88
FMI	8.88 ± 1.93	8.58 ± 1.90	8.91 ± 2.06	8.40 ± 2.40	10.54 ± 2.76	11.08 ± 2.26
FFMI	16.98 ± 1.35	16.55 ± 1.37	18.90 ± 1.89	17.60 ± 1.51***	17.69 ± 1.11	17.68 ± 1.39
FM/FFM (kg/kg^2^)	0.52 ± 0.10	0.52 ± 0.10	0.47 ± 0.10	0.47 ± 0.11	0.60 ± 0.15	0.63 ± 0.14
FM/FFM^2^ (kg/kg^2^ × 100)	1.46 ± 0.34	1.45 ± 0.32	1.01 ± 0.33	1.15 ± 0.40	1.36 ± 0.35	1.46 ± 0.34
LST (%)^‡^			57.3 ± 4.5 (55)	56.7 ± 4.8		
LST (Kg)^‡^	–	–	40.31 ± 8.65 (55)	35.56 ± 7.9**	–	–
Total fat (Kg)^‡^	–	–	28.17 ± 6.43 (62)	25.25 ± 5.44*	–	–
Total fat (%)^‡^			40.17 ± 4.28 (62)	40.50 ± 4.69		
Trunk fat (Kg)^‡^	–	–	13.95 ± 3.52 (62)	12.01 ± 2.92**	–	–
% Trunk fat^‡^	–	–	49.40 ± 3.15 (62)	47.39 ± 3.24**	–	–
**Metabolic and hormones**						
T- Chol (mg/dl)	155.6 ± 31.8	156.1 ± 31.2	143.65 ± 29.22	143.96 ± 24.18	144.7 ± 30.3	133.1 ± 19.4
LDL calc (mg/dl)	62.1 ± 32.8	53.6 ± 31.8	69.69 ± 25.13	77.40 ± 24.59	61.6 ± 24.0	62.6 ± 18.9
Insulin (μU/ml)	13.33 ± 8.38	11.42 ± 7.06	12.64 ± 8.32	10.30 ± 5.85	14.29 ± 9.14	10.45 ± 4.95
hs-CRP (mg/l)	2.68 ± 3.64	2.95 ± 4.76	2.03 ± 2.35 (48)	1.24 ± 1.42 (31)	1.10 ± 1.07 (20)	0.86 ± 1.12 (17)
Leptin (ng/ml)	21.05 ± 9.48	21.14 ± 12.13	21.39 ± 9.50 (48)	18.50 ± 7.88 (32)	24.62 ± 11.77	25.18 ± 12.25
Adiponectin (μg/ml)	12.67 ± 6.64	13.09 ± 6.00	12.77 ± 5.92 (48)	12.93 ± 4.89(32)	12.73 ± 5.99	14.50 ± 5.05
**Cardio-metabolic indices**						
HOMA-IR	2.35 ± 1.67	1.67 ± 1.62*	2.71 ± 1.81	2.13 ± 1.34	3.20 ± 2.15	2.23 ± 1.12*
TyG	8.65 ± 0.53	8.23 ± 0.34***	8.64 ± 0.56	8.28 ± 0.33***	8.73 ± 0.59	8.26 ± 0.28***
VAI	2.02 ± 1.22	1.07 ± .47***	2.11 ± 1.26	1.36 ± .47***	2.45 ± 1.23	1.42 ± 0.37***

Data are presented as means ± SD. Significant Student’s t-test *p < 0.05, **p < 0.01, ***p < 0.001 vs. the respective MUO. Body composition assessed by ^**†**^Bioimpedance (Tanita) or ^**‡**^DXA. Sample size in parentheses is indicated for incomplete data.

WC, waist circumference; p90-p75, percentile 90-percentile 75; HC, hip circumference; WhipR, waist hip ratio; WhtR, waist height ratio; FFM, fat-free mass; FMI, fat mass index; FFMI, fat-free mass index; FM, fat mass (by BIA); LST, lean soft tissue; T-Chol, total cholesterol, LDL-calc, low-density lipoprotein cholesterol; HDL, High-density lipoprotein cholesterol; hs-CRP: high-sensitivity C-reactive protein, HOMA-IR, Homeostatic Model Assessment for Insulin Resistance; TyG, triglyceride-glucose index; VAI, Visceral Adiposity Index.

Consistent with the above, age- and sex-adjusted logistic regression (Table 3) showed a 2.7- and 7.3-fold greater risk of MUO for each unit increase in age-adjusted BMI in children at T2 and T4, respectively, while elevations in absolute (but not percent) lean soft tissue, FFM, FFMI, trunk fat mass and percent trunk fat (but not total fat) also indicated MUO risk at T4. An elevated WC increased the MUO risk at T2 and T4. There were no differences in anthropometric measures at 1YPM.

**Table 3:** Adjusted (age and sex) logistic regression models of adiposity and metabolic-hormonal markers and the risk of developing MUO at Tanner 2, Tanner 4 and 1-year post-menarche in children with obesity.

	Risk of MUO [OR (95% CI)]		
	Tanner 2 n = 141	Tanner 4 n = 118	1YPM n = 59
	OR (95%CI)*	OR (95%CI)*	OR (95%CI)*
**Anthropometry**			
WC	**1.07 (1.01–1.13)**	1.07 (1.00–1.14)	1.02 (0.95–1.09)
WC ≥ percentile 90	1.28 (0.63–2.62)	1.85 (0.81–4.26)	1.05 (0.34–3.21)
WC ≥ percentile 75^$^	–	–	–
HC	1.05 (0.98–1.13)	1.07 (1.00–1.14)	1.02 (0.95–1.09)
zBMI	**2.73 (1.14–6.55)**	**7.26 (1.79–29.44)**	1.14 (0.41–3.15)
WHipR	1.40 (0.90–2.16)	1.13 (0.72–1.80)	0.94 (0.42–2.06)
WhtR	**2.04 (1.14–3.66)**	1.55 (0.82–2.94)	0.99 (0.47–2.08)
**Body composition**			
FFM (%)^†^	1.00 (0.93–1.08)	0.99 (0.91–1.08)	1.10 (0.94–1.28)
FFM (Kg)^†^	1.07 (0.99–1.16)	**1.13 (1.04–1.23)**	1.05 (0.93–1.18)
Total Fat (%)^†^	1.03 (0.95–1.12)	1.04 (0.95–1.14)	0.91 (0.78–1.06)
Total Fat (kg)^†^	1.05 (0.97–1.13)	1.05 (0.97–1.12)	0.97 (0.88–1.07)
FMI	1.17 (0.96–1.43)	1.11 (0.91–1.37)	0.88 (0.65–1.17)
FFMI	**1.46 (1.09–1.96)**	**1.69 (1.21–2.36)**	1.01 (0.66–1.53)
FM/FFM (kg/kg)	1.16 (0.72–1.88)	1.29 (0.73–2.29)	0.71 (0.35–1.42)
FM/FFM^2^ (kg/kg^2^ × 100)	0.80 (0.22–2.95)	0.56 (0.11–2.98)	0.28 (0.03–2.36)
LST (%)^‡^	–	1.00 (0.90–1.11)	–
zLST^‡^	–	**2.22 (1.02–4.85)**	–
% Total fat^‡^		1.01 (0.91–1.12)	
zTotal fat^‡^	–	1.72 (0.93–3.16)	–
% Trunk fat^‡^	–	**1.22 (1.06–1.40)**	–
zTrunk fat^‡^	–	**1.00 (1.00–1.00)**	–
**Metabolic and hormones**			
T-Chol	1.00 (0.99–1.01)	1.01 (0.99–1.03)	1.02 (1.00–1.04)
LDL Calc	1.01 (1.00–1.02)	0.99 (0.98–1.01)	1.00 (0.97–1.02)
Insulin	1.05 (1.00–1.10)	1.06 (1.00–1.13)	1.10 (1.00–1.21)
hsCRP	0.98 (0.91–1.07)	**1.36 (1.02–1.82)**^§^	1.20 (0.62–2.31)^§§^
Leptin	1.00 (0.97–1.03)	1.05 (0.99–1.11)^§^	1.00 (0.95–1.06)
Adiponectin	0.98 (0.93–1.04)	0.99 (0.91–1.08)^§^	0.94 (0.85–1.04)
**Cardio-metabolic indices**			
HOMA	**1.38 (1.09–1.76)**	**1.35 (1.01–1.79)**	**1.60 (1.02–2.50)**
TyG	**8.96 (3.49–23.00)**	**6.11 (2.41–15.48)**	**6.11 (2.41–15.48)**
VAI	**7.70 (3.50–16.96)**	**5.51 (2.56–11.84)**	**6.60 (2.07–21.07)**

*OR adjusted for chronological age and sex (except in 1YPM, only age-adjusted). MUO, metabolically unhealthy obesity; OR, odds ratio; CI, confidence interval. Body composition assessed by ^**†**^Bioimpedance (Tanita) or ^**‡**^DXA. WC, waist circumference; HC: hip circumference; WHipR, waist hip ratio, WhtR, waist height ratio; FFM, fat-free mass; FM, fat mass (by BIA); FMI, fat mass index; FFMI, fat-free mass index; LST: lean soft tissue. ^§^n = 80. ^§§^n = 37. ^$^There was only one subject in the entire sample that did not present WC ≥ percentile 75. Values in bold indicate statistical significance.

#### Metabolic and endocrine variables and indices

MHO subjects showed lower HOMA-IR index at T2 and 1 YPM while TyG and VAI were lower for this group at all pubertal stages (Table 2). Adjusted logistic regression models were consistent with these results (Table 3) and also showed that the proinflammatory marker hs-CRP was significantly associated with MUO risk by 36% at T4, as was T-Chol by 2% at 1YPM.

### Metabolically healthy and unhealthy children without obesity

#### Anthropometric and body composition variables

The differences between MUNO and MHNO phenotypes are shown in Table 4. At T2, we observed that healthy subjects had a slightly lower WhipR and FFMI; while at T4 all anthropometric parameters were lower in MHNO compared to MUNO, with the exception of % lean soft tissue and FFM which were greater in MHNO. At the 1YPM assessment WC, BAZ, WhtR, FMI and % fat were lower and % FFM was higher in MHNO than MUNO. Notably, in contrast with the results observed in subjects with obesity, we found higher values of FM/FFM in the unhealthy phenotypes at T4 and 1YPM, which was also the case for FM/FFM^2^ at 1YPM. Regression analysis confirmed these results, with an eight-fold increase in the risk for unhealthy phenotype at 1YPM with every point increase in FM/FFM^2^. FM/FFM also was associated with significant elevations in odds ratio for presenting the unhealthy phenotype at T4 and 1YPM (1.8 and 2.0-fold, respectively). Central obesity based on age- and sex- adjusted WC ≥ 90th percentile was present in less than 5% of the MUNO/MHNO subjects. Interestingly, when those in the WC ≥ 75th percentile were analyzed, the proportion was greater in unhealthy vs healthy subjects at T4 and 1YPM (T2 showed a strong trend at p = 0.05). Consistent results were observed in the adjusted models (Table 5), with an approximate 30% higher risk for the unhealthy phenotype for each unit increase in WhipR at T2, and elevations in all anthropometric and body composition parameters (and decrease in % FFM and lean soft tissue) considerably increasing risk for the unhealthy phenotype at T4. For girls assessed at the 1YPM visit, higher WC, zBMI, WhtR, FMI, fat percentage and mass, as well as lower FFM percentage were associated with elevated risk for being unhealthy.

**Table 4:** Description of adiposity, metabolic and hormonal markers in participants of the GOCS Study without obesity at Tanner 2, Tanner 4 and 1 year post menarche.

	Tanner 2		Tanner 4		1 year post menarche	
	MUNO (199)	MHNO (289)	MUNO (227)	MHNO (372)	MUNO (87)	MHNO (200)
**Anthropometry**						
WC (cm)	67.53 ± 6.95	66.46 ± 6.80	72.96 ± 7.24	70.01 ± 6.52***	73.7 ± 6.5	71.8 ± 6.6*
WC ≥ p90 (n)	6 (3.0%)	3 (1.0%)	10 (4.4%)	8 (2.2%)	4 (4.6%)	2 (1.0%)
WC ≥ p75 (n)	50 (25.1%)	52 (18.0%)	74 (32.6%)	72 (19.3%)^**§§§**^	29 (33.3%)	43 (21.5%)^**§**^
HC (cm)	77.92 ± 5.80	77.55 ± 6.22	86.13 ± 6.13	83.97 ± 5.91***	90.7 ± 5.4	89.4 ± 6.1
zBMI	0.64 ± 0.91	0.47 ± 0.94	0.72 ± 0.93	0.42 ± 0.95***	0.82 ± 0.78	0.53 ± 0.88**
WHipR	0.87 ± 0.05	0.86 ± 0.04*	0.85 ± 0.04	0.83 ± 0.04***	0.81 ± 0.04	0.80 ± 0.04
WhtR	0.47 ± 0.04	0.47 ± 0.04	0.47 ± 0.04	0.45 ± 0.04***	0.47 ± 0.04	0.46 ± 0.04**
**Body composition**						
FFM (%)^†^	76.5 ± 4.7	76.4 ± 4.5	76.9 ± 5.4	77.9 ± 6.4*	69.9 ± 5.6	71.5 ± 5.1*
FFM (Kg)^†^	29.25 ± 4.61	28.65 ± 5.15	38.79 ± 7.27	37.09 ± 7.44**	36.18 ± 3.98	36.28 ± 4.57
Total Fat (%)^†^	23.52 ± 4.66	23.62 ± 4.34	23.13 ± 5.38	21.83 ± 5.18**	29.66 ± 4.28	28.29 ± 3.87**
Total Fat (kg)^†^	9.17 ± 2.92	9.03 ± 2.88	11.81 ± 3.88	10.48 ± 3.46***	15.66 ± 4.05	14.72 ± 3.84
FMI	4.50 ± 1.34	4.42 ± 1.27	4.80 ± 1.51	4.35 ± 1.44***	6.40 ± 1.50	5.96 ± 1.46*
FFMI	14.29 ± 1.21	13.98 ± 1.33*	15.62 ± 1.58	15.15 ± 1.72***	14.85 ± 1.38	14.71 ± 1.48
FM/FFM (kg/kg)	0.31 ± 0.08	0.31 ± 0.08	0.31 ± 0.09	0.29 ± 0.09**	0.43 ± 0.09	0.40 ± 0.08**
FM/FFM^2^ (kg/kg^2^ × 100)	1.09 ± 0.31	1.12 ± 0.31	0.83 ± 0.32	0.81 ± 0.32	1.21 ± 0.35	1.10 ± 0.20**
LST (%)^‡^	–	–	67.2 ± 7.4 (177)	68.7 ± 7.2* (300)	–	–
LST (Kg)^‡^	–	–	33.54 ± 6.96 (177)	32.45 ± 7.16 (300)	–	–
Total fat (%)^‡^			30.45 ± 7.15 (198)	28.87 ± 7.24* (329)		
Total fat (Kg)^‡^	–	–	15.23 ± 4.88 (n = 198)	13.50 ± 4.39*** = 330)	–	–
Trunk fat (Kg)^‡^	–	–	6.54 ± 2.70 (198)	5.69 ± 2.43*** (330)	–	–
% Trunk fat^‡^	–	–	41.8 ± 5.0 (198)	41.0 ± 4.9 (330)	–	–
**Metabolic and hormones**						
T-Chol (mg/dl)	149.5 ± 27.4	150.8 ± 24.6	135.5 ± 26.8	140.8 ± 23.9*	136.0 ± 30.1	135.4 ± 22.6
LDL calc (mg/dl)	59.7 ± 29.5	62.0 ± 28.2	68.1 ± 26.3	70.6 ± 25.0	69.9 ± 21.6	65.7 ± 20.8
Insulin (μU/ml)	10.37 ± 6.32	9.44 ± 5.14	10.38 ± 5.50	10.34 ± 5.60	11.8 ± 7.0	11.4 ± 5.7
hs-CRP (mg/dl)	1.69 ± 2.76	1.83 ± 2.69	1.37 ± 1.91 (138)	1.35 ± 2.22 (256)	1.18 ± 1.96 (50)	0.78 ± 1.27 (99)
Leptin (ng/ml)	11.60 ± 7.11	10.96 ± 6.16	13.24 ± 8.05 (139)	12.32 ± 7.79 (n = 256)	18.1 ± 9.7	19.5 ± 9.1
Adiponectin (μg/ml)	13.51 ± 6.51	14.47 ± 6.44	15.00 ± 6.03 (139)	14.40 ± 5.95 (256)	15.4 ± 6.2	16.0 ± 6.8
**Cardio-metabolic indices**						
HOMA	1.79 ± 1.50	1.63 ± 1.17	2.12 ± 1.28	2.03 ± 1.22	2.52 ± 1.57	2.38 ± 1.28
TyG	8.39 ± 0.50	8.13 ± 0.37***	8.47 ± 0.55	8.20 ± 0.32***	8.38 ± 0.51	8.09 ± 0.33***
VAI	1.51 ± 0.90	0.97 ± 0.42***	1.78 ± 0.84	1.10 ± 0.42***	1.97 ± 0.72	1.17 ± 0.41***

Data are presented as means ± SD. Significant Student’s t-test *p < 0.05, **p < 0.01, ***p < 0.001 vs. the respective MUNO. Significant Pearson Chi2 test ^§^p < 0.05, ^§§§^p < 0.001 vs. MUNO.

Body composition assessed by ^†^Bioimpedance (Tanita) or ^**‡**^DXA. Sample size in parentheses is indicated for incomplete data. WC, waist circumference; p90-p75, percentile 90-percentile 75; HC, hip circumference; WhipR, waist hip ratio; WhtR, waist height ratio; FFM: fat-free mass; FMI, fat mass index; FFMI, fat-free mass index; FM, fat mass (by BIA); LST, lean soft tissue; T-Chol, total cholesterol, LDL-calc, low-density lipoprotein cholesterol; HDL, High-density lipoprotein cholesterol; hsCRP, high-sensitivity C-reactive protein, HOMA-IR, Homeostatic Model Assessment for Insulin Resistance; TyG, triglyceride-glucose index; VAI, Visceral Adiposity Index.

**Table 5:** Adjusted (age and sex) logistic regression models of adiposity and metabolic-hormonal markers and the risk of developing MUNO at Tanner 2, Tanner 4 and 1-year post-menarche in children without obesity.

	Risk of MUNO [OR (95% CI)]		
	Tanner 2 n = 488	Tanner 4 n = 599	1YPM n = 287
	OR (95%CI)*	OR (95%CI)*	OR (95%CI)*
**Anthropometry**			
WC	1.02 (1.00–1.05)	**1.06 (1.04–1.09)**	**1.04 (1.00–1.08)**
WC ≥ percentile 90	3.12 (0.77–12.68)	**2.30 (0.88–6.02)**	4.55 (0.81–25.56)
WC ≥ percentile 75	**1.56 (1.00–2.43)**	**2.18 (1.47–3-22)**	1.68 (0.95–2.99)
HC	1.01 (0.98–1.05)	**1.06 (1.03–1.09)**	1.04 (0.99–1.09)
zBMI	1.16 (0.95–1.43)	**1.48 (1.22–1.80)**	**1.46 (1.04–2.04)**
WHipR	**1.30 (1.03–1.64)**	**1.48 (1.19–1.84)**	1.35 (0.97–1.87)
WhtR	1.28 (0.98–1.66)	**1.70 (1.33–2.16)**	**1.65 (1.11–2.43)**
**Body composition**			
FFM (%)^†^	1.00 (0.96–1.04)	**0.95 (0.92–0.99)**	**0.94 (0.89–0.99)**
FFM (Kg)^†^	1.04 (0.98–1.09)	**1.06 (1.02–1.10)**	1.00 (0.94–1.06)
Total Fat (%)^†^	1.00 (0.96–1.05)	**1.08 (1.04–1.12)**	**1.10 (1.03–1.17)**
Total Fat (kg)^†^	1.02 (0.96–1.09)	**1.10 (1.05–1.16)**	**1.07 (1.00–1.15)**
FMI	1.06 (0.92–1.22)	**1.28 (1.14–1.45)**	**1.24 (1.04–1.47)**
FFMI	**1.22 (1.04–1.45)**	**1.26 (1.11–1.43)**	1.06 (0.88–1.27)
zFM/FFM (kg/kg)	1.04 (0.74–1.46)	**1.81 (1.32–2.47)**	**1.99 (1.28–3.09)**
zFM/FFM^2^ (kg/kg^2^ × 100)	0.69 (0.27–1.76)	2.71 (0.98–7.47)	**8.47 (2.04–35.13)**
LST (%)^‡^		**0.96 (0.93–0.99)**	
zLST^‡^	–	**1.47 (1.01–2.13)**	–
% Total fat^‡^		**1.05 (1.02–1.08)**	
zTotal fat^‡^	–	**1.73 (1.32–2.26)**	–
% Trunk fat^‡^	–	1.04 (1.00–1.07)	–
zTrunk fat^‡^	–	**1.64 (1.25–2.15)**	–
**Metabolic and hormones**			
T-Chol	1.00 (0.99–1.01)	**0.99 (0.98–1.00)**	1.00 (0.99–1.01)
LDL Calc	1.00 (0.99–1.00)	1.00 (0.99–1.00)	1.01 (1.00–1.02)
Insulin	1.03 (1.00–1.07)	1.00 (0.97–1.03)	1.00 (0.96–1.05)
CRP	0.98 (0.91–1.05)	1.00 (0.91–1.10)^§^	1.22 (0.96–1.55)^§§^
Leptin	1.02 (0.99–1.05)	1.01 (0.99–1.04)^§^	0.99 (0.96–1.02)
Adiponectin	0.97 (0.94–1.00)	1.01 (0.98–1.05)^§^	1.00 (0.96–1.04)
**Cardio-metabolic indices**			
HOMA	1.11 (0.96–1.27)	1.05 (0.92–1.20)	1.04 (0.86–1.25)
TyG	**4.07 (2.58–6.43)**	**4.71 (3.03–7.31)**	**5.63 (2.83–11.17)**
VAI	**6.74 (4.28–10.60)**	**11.76 (7.46–18.54)**	**14.82 (7.43–29.52)**

*OR adjusted for chronological age and sex (except in 1YPM, only age-adjusted). MUNO, metabolically unhealthy no-obesity; OR, odds ratio; CI, confidence interval. Body composition assessed by ^†^Bioimpedance (Tanita) or ^‡^DXA (n = 528). WC, waist circumference; HC, hip circumference; WHipR, waist hip ratio, WhtR, waist height ratio; FFM, fat-free mass; FM, fat mass (by BIA); FMI, fat mass index; FFMI, fat-free mass index; LST, lean soft tissue. ^§^n = 395, ^§§^n = 149. Values in bold indicate statistical significance.

#### Metabolic and endocrine variables and indices

Among metabolic parameters in children without obesity, T-Chol had a small protective effect against unhealthy phenotype at T4 (Table 5), which could be driven by the lower HDL-C levels found in the MUNO group (Table S3). VAI and TyG levels were lower in the healthy phenotype at all stages, whereas there were no differences in HOMA-IR between phenotypes in children without obesity.

## Discussion

The present study evaluated whether the consensus definition for MHO proposed by Damanhoury et al.^12^ is applicable and consistent with body composition and metabolic risk variables in Chilean children at pubertal stages T2, T4 and girls 1YPM. More MUNO children were defined as “unhealthy” with only one alteration, as opposed to MUO children allocated to the “unhealthy” group with 2 or more altered factors. Nevertheless, the MUNO phenotype was indeed associated with greater metabolic risk, which highlights the relevance of detecting this group. The most frequently altered factors among MUO were HDL, TG and SBP, while low HDL-C was notably more frequent among MUNO. Body composition at T4 revealed more central adiposity in MUO as compared with MHO. Also the MUO participants had worse metabolic indicators such as HOMA (except for T4), TyG and VAI. Interestingly, among children without obesity at T4 and 1YPM, the MUNO group had lower % FFM and greater FMI and % fat, which was more centrally distributed, with higher TyG and VAI. The percentage of MHO in our population of children with obesity was 52%, 39% and 49% for T2, T4 and the subsample of girls at 1YPM, respectively, which is consistent with previous studies^35,36^. Importantly, our observations revealed that “unhealthy” subjects among children without obesity was 41% (T2), 38% (T4) and 30% (1YPM), which should be a reason for concern.

Central fat accumulation in childhood into adolescence has been directly associated with unfavorable cardiometabolic health marker profiles, regardless of total body fat^37^. Our results indicate that at T2, HOMA-IR was directly correlated with WC but not with zBMI, in children classified as metabolically-unhealthy (data not shown), consistent with a more relevant role of central than whole body fatness inducing metabolic derangements. The application of the consensus equation at T4, where we had fat distribution data available, was consistent with higher trunk fat present in unhealthy phenotypes (MUO and MUNO), which confirms the ability of this equation to reflect the role of central fat distribution in the metabolic risk, independent of obesity. When analyzing the the four phenotypes together, central obesity based on age- and sex-adjusted WC^21^ was significantly associated with the unhealthy phenotype (spearman chi^2^ p < 0.05, < 0.001 and < 0.01 for T2, T4 and 1YPM, respectively, data not shown). Our study shows that a higher WhtR is associated with higher risk for MUO at T2, when adjusting for age and sex. Waist alone became a significant predictor at T4. Interestingly, these ratios were consistently more relevant in subjects without obesity; at T2 and T4, higher WhipR elevated the risk for the unhealthy phenotype. WhtR showed 70% and 65% elevations in risk per unit increase at T4 and 1YPM, respectively, which is consistent with previous reports of WhtR as a predictor of metabolic syndrome in a Chilean adolescent cohort^38^.

An additional parameter used to assess obesity-related metabolic risk is VAI, a good estimator of visceral adiposity^39^ associated with insulin resistance, diabetes, metabolic syndrome and cardio- and cerebrovascular events in different adult and pediatric populations.^31,40–43^ As expected given common components in VAI and Damanhoury’s equation, VAI was highly consistent with the MHO-MUO definition. Importantly, Ejtahed et al.^42^ established in a group aged 7–18 years (both sexes combined) a VAI cutoff value of 1.58 to detect metabolic syndrome, which agrees with our results at T2 where MUO show VAI values above (2.02) and MHO below (1.07) the cutoff. Interestingly, in both T4 and 1YPM the MUNO phenotype VAI surpassed the cutoff associated with metabolic syndrome^42,43^, indicating that this is valid also for children without obesity. More recently, Vissuzo et al.^43^ defined a similar VAI cutoff value (1.775) to identify metabolic syndrome, closely consistent with our findings as well. These authors established that VAI was correlated with BMI, WhtR, HOMA-IR, systolic blood pressure, LDL, HDL and TG, which was also the case in our study (data not shown). This supports the clinical validity of the consensus equation and, furthermore, the use of MHO-MUO-MHNO-MUNO classification as a tool for assessing metabolic risk and therapeutic priorities in children even if not at an obvious risk due to having obesity.

Other available indices for metabolic risk are TyG and HOMA-IR, representing skeletal muscle- and liver-induced insulin resistance, respectively^33^. Elevated TyG was consistent with higher odds for the unhealthy phenotype both in children with and without obesity. TyG values were lower in MHNO children at all stages, and each unit increase elevated by four to five-fold the risk for the unhealthy phenotype. Moreover, at T2 healthy obese vs healthy nonobese subjects did not differ in TyG, consistent with its specificity as a marker of metabolic risk independent of BMI^44^. On the other hand, mean values for HOMA-IR in MUO and MHO children at T2 were consistent with established cutoff values of ≥ 2 defining insulin resistance children at this pubertal stage^45^. Elevated values of HOMA-IR were consistent with higher odds for MUO allocation, but this was not the case for MUNO. Nevertheless, HOMA-IR was consistently higher in metabolically unhealthy children, revealing phenotype information may be more relevant than obesity alone and further supporting the consensus definition as a means for assessing this relevant risk at young ages.

Unhealthy obesity has been widely linked to a low-grade inflammatory state. Our observations in children at T4 were consistent associating hs-CRP with higher MUO risk. At T2, there was a significant direct correlation of hs-CRP with zBMI and WC only in the unhealthy phenotype (data not shown). Unfortunately, we had many missing values for this parameter, which may have precluded us from establishing more associations, however these data are indicative of the value of phenotype allocation detecting underlying differences in this relevant inflammatory status indicator.

The relevance of focusing on children and adolescents showing the MUNO phenotype has recently been underscored. A 2021 meta-analysis^15^ concluded that the prevalence of the so-called “normal weight obese” phenotype in adolescents ranges from 7 to 56%, consistent with our data (30–41%). The authors highlighted the relevance of detecting and treating these subjects at this young age, given the elevated risk for future disease development. Interestingly, only in children without obesity, we observed higher values of FM/FFM and FM/FFM^2^ in the unhealthy phenotype. Higher values of this fat to lean mass or “load capacity model” have been particularly associated to a “thin-fat-owerweight” or “sarcopenic obese” phenotype^22^, thus these results further support the relevance of using the Damanhoury formula to assess risk in children without obesity. Importantly, a study in 9–11 years-old children^46^ revealed lower cardio-respiratory and muscular fitness in children with normal zBMI but high body fat as compared with their normal-weight peers with normal fat levels, and physical fitness deficits that were similar to their overweight and obese peers. The higher body fat and lower lean mass percent in our unhealthy phenotypes allows us to speculate that we would observe the same trends in physical performance in our population.

Our study is not exempt of limitations. We did not have complete data of blood pressure at T2, thus we had larger number of children that were not possible to categorize in phenotypes. We started measuring this parameter later in the cohort, thus the earlier children at T2 were not included (predominantly in girls) and the excluded group had a younger age, albeit their BMI did not differ. Also our results are not generalizable to all infant population, because we included only children of normal birthweight and from full term pregnancy (premature were excluded). Among the strengths of this study, all anthropometric measurements were performed by trained dietitians and we have a complete dataset of body composition beyond BMI, which allowed us for a more thorough assessment.

In summary, this study contributes to assess the validity and predictive usefulness of Damanhoury’s model to define MHO/MUO and extended to children without obesity. Importantly, a higher percentage of children without obesity were classified as metabolically unhealthy with only one altered parameter. A less strict formula tolerating one alteration, as others have suggested, would have excluded these children from the unhealthy category. Given that MUNO children were associated with unfavorable conditions, our observations support that the definition of healthy phenotypes should continue to require no altered parameters.. We suggest that the consensus equation proposed by Damanhoury et al.^12^ can also be used for children without obesity in order to assess risk in this less-studied population. The concept of metabolic health in children and its early assessment may have an impact on future health risk.

## Supplementary Information

Supplementary Information.

## Data Availability

The datasets used and/or analysed during the current study available from the corresponding author on reasonable request.
