# Supplementary material for: Assessment of a consensus definition of obesity and metabolic health phenotypes in children at different pubertal stages
Source: Sci Rep. 2022 Dec 7;12:21129. doi: 10.1038/s41598-022-25771-5 (PMC9729631; doi:10.1038/s41598-022-25771-5)
Supplement: Supplementary file 1 — Supplementary Information. [file 41598_2022_25771_MOESM1_ESM.docx]

**Assessment of a consensus definition of obesity and metabolic health phenotypes in children at different pubertal stages:**

Ana Pereira ^1^, Marcela Reyes ^1^, Camila Corvalán ^1^, Juan Pablo Espejo ^1^, Verónica Mericq ^2^, Mariana Cifuentes^1,3^ *.

*Corresponding author: Mariana Cifuentes, Instituto de Nutrición y Tecnología de los Alimentos, Universidad de Chile. El Libano 5524 Macul, Santiago Chile; mcifuentes@inta.uchile.cl

**Affiliations**

^1^Institute of Nutrition and Food Technology (INTA), Universidad de Chile, Santiago, Chile.

^2^Institute of Maternal and Child Research, Faculty of Medicine, University of Chile.

^3^Advanced Center for Chronic Diseases (ACCDiS), Santiago, Chile.

**SUPPLEMENTARY INFORMATION**

**
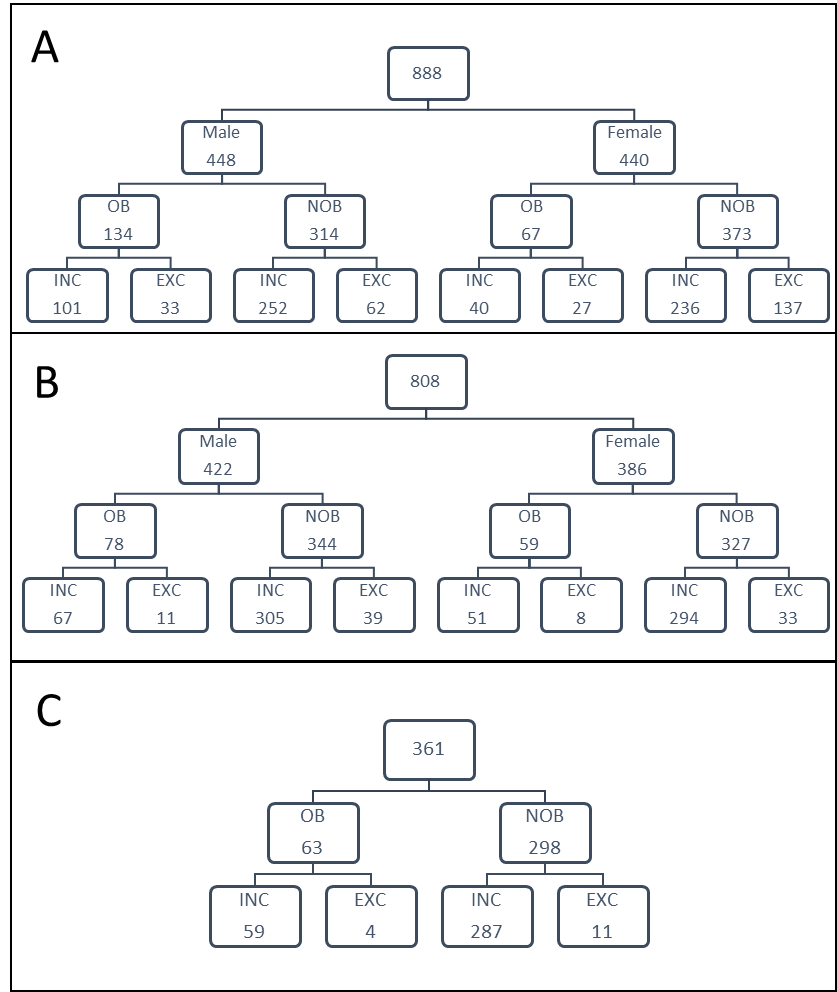
**

**Figure S1.** Diagram showing the number of participants included (INC) and excluded (EXC) from the analysis by nutritional status (with obesity [OB] or without obesity [NOB]) and sex, for A) Tanner 2; B) Tanner 4; and C) One year post-menarche stages.


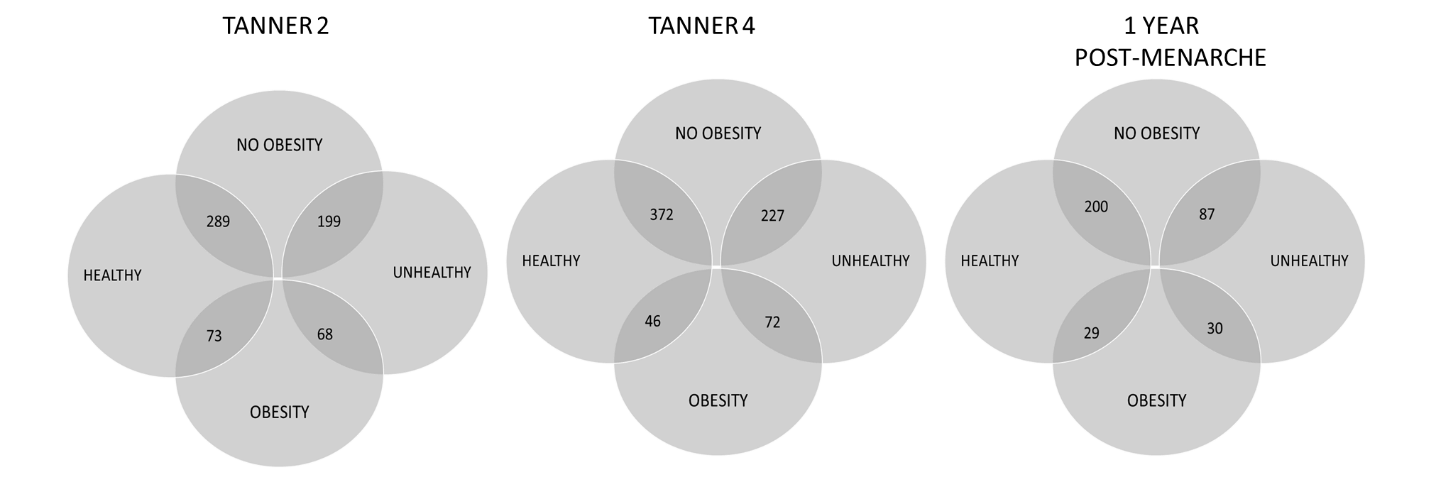


**Figure S2**. Venn diagram of the number of participants in each phenotype at each Tanner stage

**TABLE S1: Variables in children with (OB) and without obesity (NOb) that were excluded from the study due to incomplete available data at Tanner 2, Tanner 4 and one-year post-menarche (1YPM)**

|  | **Tanner 2** | | | | **Tanner 4** | | | | **1yPM** | | | |
| --- | --- | --- | --- | --- | --- | --- | --- | --- | --- | --- | --- | --- |
|  | OB | | **NOb** | | **OB** | | **NOb** | | **OB** | | **NOb** | |
|  | I (141) | E (60) | I (488) | E (199) | I (118) | E (19) | I (599) | E (72) | I (59) | E (4) | I (287) | E (11) |
| Males (%) | 101 (72) | 33 (55) **^†^** | 252 (52) | 62 (31) **^†††^** | 67 (57) | 11 (58) | 305 (51) | 39 (54) | __ | __ | __ | __ |
| Age | 11.0±1.1 | 10.2±2.1** | 10.9±1.2 | 9.7±1.9*** | 12.4±1.6 | 12.7±1.6 | 12.5±1.5 | 12.8±14 | 12.8±1.1 | 14.0±1.3* | 13.0±1.0 | 14.5±1.4*** |
| zBMI | 2.54±0.42 | 2.51±0.45 | 0.54±0.93 | 0.65±0.97 | 2.43±0.37 | 2.30±0.28 | 0.53±0.94 | 0.49±1.00 | 2.46±0.07 | 2.36±0.34 | 0.61±0.87 | 0.58±1.13 |

I= included; E= excluded from the study due to incomplete data. zBMI: body mass index standardized for age. Significantly different from the respective “included” group *P<0.05, **p<0.01, ***p<0.001, Student’s t-test, **^†^**P<0.05, **^††^**p<0.01, **^†††^**p<0.001, Pearson Chi2.

**TABLE S2. Mean values of the parameters used in Damanhoury’s definition of metabolic health across phenotypes in Tanner 2**

| Tanner 2 | MHO | MUO | MHNO | MUNO | p-value* | | |
| --- | --- | --- | --- | --- | --- | --- | --- |
|  | **(n=73)** | **(n=68)** | **(n=289)** | **(n=199)** | Obesity | Health | Obe x  Health |
| Weight | 55.4±10.3 | 55.4±10.2 | 37.67±7.30 | 38.39±6.71 | 0.0000 | 0.6654 | 0.6050 |
| Height | 147.7±8.8 | 146.0±7.4 | 142.59±8.09 | 142.68±7.58 | 0.0000 | 0.2897 | 0.2422 |
| zBMI | 2.46±0.36 | 2.63±0.47 | 0.47±0.94 | 0.64±0.91 | 0.0000 | 0.0385 | 0.9689 |
| SBP | 102.2±6.7 | 106.0±8.5 | 97.4±6.8 | 101.8±9.5 | 0.0000 | 0.0000 | 0.6430 |
| DBP | 58.7±4.4 | 60.4±6.2 | 54.1±5.5 | 55.6±6.7 | 0.0000 | 0.0058 | 0.9082 |
| TG | 89.4±28.3^a^ | 142.3±77.5^b^ | 82.1±28.5^a^ | 109.0±57.7^c^ | 0.0000 | 0.0000 | **0.0038** |
| HDL | 52.6±8.7 | 45.6±11.0 | 53.4±8.7 | 45.0±11.0 | 0.9093 | 0.0000 | 0.4771 |
| Glycemia | 88.8±6.5 | 92.0±8.6 | 88.2±6.2 | 91.3±8.6 | 0.3773 | 0.0000 | 0.9604 |

*2-way Anova, obesity and metabolic health (“health”) as independent variables. Values with different letters represent significant differences (Bonferroni post-hoc pairwise analysis performed upon a significant interaction effect). MHO: metabolically healthy obesity; MUO: metabolically uhealthy obesity; MHNO: metabolically healthy-no obesity; MUNO: metabolically healthy-no obesity; zBMI: body mass index standardized for age; SPB: systolic blood pressure (mmHg); DBP: diastolic blood pressure (mmHg); TG: triglycerides (mg/dL); HDL: high-density lipoprotein (mg/dL); glycemia (mg/dL).

**TABLE S3. Mean values of the parameters used in Damanhoury’s definition of metabolic health across phenotypes in Tanner 4**

| Tanner 4 | MHO | MUO | MHNO | MUNO | p-value* | | |
| --- | --- | --- | --- | --- | --- | --- | --- |
|  | (n=46) | (n=72) | (n=372) | (n=227) | Obesity | Health | Obe x  Health |
| Weight | 63.20±11.90^a^ | 71.95±14.54^b^ | 47.64±9.05^c^ | 50.59±9.58^d^ | 0.0000 | 0.0000 | **0.0055** |
| Height | 155.21±10.62 | 159.91±9.58 | 155.7±10.0 | 156.9±9.0 | 0.2131 | 0.0036 | 0.0766 |
| zBMI | 2.30±0.22 | 2.51±0.42 | 0.42±0.95 | 0.72±0.93 | 0.0000 | 0.0054 | 0.6552 |
| SBP | 103.04±7.06^ac^ | 111.72±10.15^b^ | 100.80±8.02^a^ | 104.64±9.96^c^ | 0.0000 | 0.0000 | **0.0083** |
| DBP | 58.0±4.8 | 60.8±6.8 | 54.7±5.7 | 56.6±6.1 | 0.0000 | 0.0002 | 0.4382 |
| TG | 96.96±27.95 | 147.07±76.87 | 89.33±26.66 | 123.04±62.69 | 0.0013 | 0.0000 | 0.0949 |
| HDL | 49.22±6.40^ab^ | 46.00±10.11^ac^ | 51.90±7.76^b^ | 43.81±11.58^c^ | 0.7980 | 0.0000 | **0.0115** |
| Glycemia | 85.37±6.01 | 88.81±7.78 | 85.80±6.91 | 88.45±11.24 | 0.9710 | 0.0006 | 0.6548 |

*2-way Anova, obesity and metabolic health (“health”) as independent variables. Values with different letters represent significant differences (Bonferroni post-hoc pairwise analysis performed upon a significant interaction effect, indicated by the interaction P value in bold). MHO: metabolically healthy obesity; MUO: metabolically uhealthy obesity; MHNO: metabolically healthy-no obesity; MUNO: metabolically healthy-no obesity; zBMI: body mass index standardized for age; SPB: systolic blood pressure (mmHg); DBP: diastolic blood pressure (mmHg); TG: triglycerides (mg/dL); HDL: high-density lipoprotein (mg/dL); glycemia (mg/dL).

**TABLE S4. Age, sex and parameters used in Damanhoury’s definition of metabolic health across phenotypes in girls 1-year post menarche.**

| 1YPM | MHO | MUO | MHNO | MUNO | p-value* | | |
| --- | --- | --- | --- | --- | --- | --- | --- |
|  | (n=29) | (n=30) | (n=200) | (n=87) | Obesity | Health | Obe*  Health |
| Weight | 70.9±10.0 | 73.0±15.8 | 50.97±7.37 | 52.07±6.93 | 0.0000 | 0.1950 | 0.6773 |
| Height | 156.7±6.4 | 158.3±6.4 | 156.9±5.9 | 156.1±5.4 | 0.2706 | 0.6570 | 0.1528 |
| zBMI | 2.44±0.31 | 2.48±0.68 | 0.53±0.88 | 0.82±0.78 | 0.0000 | 0.1752 | 0.2795 |
| SBP | 101.08±9.38^a^ | 110.79±8.79^b^ | 100.10±7.38^a^ | 101.76±9.54^a^ | 0.0000 | 0.0000 | **0.0009** |
| DBP | 58.5±6.1 | 61.2±6.2 | 56.2±5.8 | 57.0±6.7 | 0.0003 | 0.0512 | 0.3029 |
| TG | 93.28±24.21^ac^ | 159.07±82.29^b^ | 80.91±25.45^a^ | 113.39±59.97^c^ | 0.0000 | 0.0000 | **0.0089** |
| HDL | 51.93±5.97^a^ | 51.33±11.92^a^ | 52.95±7.97^a^ | 43.03±13.08^b^ | 0.0105 | 0.0002 | **0.0011** |
| Glycemia | 85.86±7.05 | 90.43±6.78 | 84.74±6.89 | 86.44±8.31 | 0.0159 | 0.0032 | 0.1745 |

*2-way Anova, obesity and metabolic health as independent variables. Values with different letters represent significant differences (Bonferroni post-hoc pairwise analysis performed upon a significant interaction effect, indicated by the interaction P value in bold). MHO: metabolically healthy obesity; MUO: metabolically uhealthy obesity; MHNO: metabolically healthy-no obesity; MUNO: metabolically healthy-no obesity; zBMI: body mass index standardized for age; SPB: systolic blood pressure (mmHg); DBP: diastolic blood pressure (mmHg); TG: triglycerides (mg/dL); HDL: high-density lipoprotein (mg/dL); glycemia (mg/dL).
